# The Growing Importance of CNVs: New Insights for Detection and Clinical Interpretation

**DOI:** 10.3389/fgene.2013.00092

**Published:** 2013-05-30

**Authors:** Armand Valsesia, Aurélien Macé, Sébastien Jacquemont, Jacques S. Beckmann, Zoltán Kutalik

**Affiliations:** ^1^Genetics Core, Nestlé Institute of Health SciencesLausanne, Switzerland; ^2^Department of Medical Genetics, University of LausanneSwitzerland; ^3^Swiss Institute of BioinformaticsLausanne, Switzerland; ^4^Service of Medical Genetics, Centre Hospitalier Universitaire VaudoisLausanne, Switzerland; ^5^Institute of Social and Preventive Medicine, Centre Hospitalier Universitaire VaudoisLausanne, Switzerland

**Keywords:** copy number variation, genome-wide association studies, personalized medicine, sequencing, complex disease, genomics, bioinformatics

## Abstract

Differences between genomes can be due to single nucleotide variants, translocations, inversions, and copy number variants (CNVs, gain or loss of DNA). The latter can range from sub-microscopic events to complete chromosomal aneuploidies. Small CNVs are often benign but those larger than 500 kb are strongly associated with morbid consequences such as developmental disorders and cancer. Detecting CNVs within and between populations is essential to better understand the plasticity of our genome and to elucidate its possible contribution to disease. Hence there is a need for better-tailored and more robust tools for the detection and genome-wide analyses of CNVs. While a link between a given CNV and a disease may have often been established, the relative CNV contribution to disease progression and impact on drug response is not necessarily understood. In this review we discuss the progress, challenges, and limitations that occur at different stages of CNV analysis from the detection (using DNA microarrays and next-generation sequencing) and identification of recurrent CNVs to the association with phenotypes. We emphasize the importance of germline CNVs and propose strategies to aid clinicians to better interpret structural variations and assess their clinical implications.

## Background Information on CNVs

Genetic variations in the human genome take many forms ranging from large chromosomal anomalies (segmental aneuploidy) to single nucleotide variant (SNVs). Deletion, insertion, and duplication events which give rise to copy number variations (CNVs) have been found genome-wide in humans (Iafrate et al., 2004; Sharp et al., 2005; Feuk et al., 2006; Fiegler et al., 2006; Freeman et al., 2006; Redon et al., 2006; Kidd et al., 2008, 2010; Perry et al., 2008; Conrad et al., 2010; Valsesia et al., 2012) and other species (Dopman and Hartl, 2007; Graubert et al., 2007; Guryev et al., 2008; Lee et al., 2008; Fontanesi et al., 2010; Liu et al., 2010). CNVs are classically defined as events longer than 1 kb (Feuk et al., 2006); smaller events are referred to as indels (see additional definitions in Box 1). With the advent of next-generation sequencing (NGS), CNVs as small as 500 bp can be identified. CNVs can occur at different frequencies in a given population. When this frequency is greater than 1%, the CNV is referred to as a copy number polymorphism (CNP) (Feuk et al., 2006) (Box 1). This contrasts with single nucleotide polymorphisms (SNPs) whose frequencies are by definition greater than 1%.

Box 1 **Additional definitions.**

**Structural variants**

Structural variation defines a large class of genomic alterations. These alterations can be quantitative (copy number variants, indels), positional (translocations), or orientational (inversions). This term is used in a neutral sense and nothing is suggested with regards to variation frequency or to association with a phenotype/disease.

**Single Nucleotide Polymorphism (SNP)**

Single nucleotide polymorphisms are the most common type of DNA polymorphisms, which occur when a single nucleotide in the genome sequence is altered. By definition, SNPs occur in a population with a frequency greater than 1%. When this frequency criterion is not met, this variation is referred to as a single nucleotide variant (SNV).

**Copy Number Variant (CNV)**

Copy number variant refers to a segment of DNA, for which copy number differences can be observed between individuals. Translocations and inversions do not involve copy number changes and thus are not considered as copy number variants. Following the initial genome-wide discovery of CNVs using BAC arrays and early SNP arrays, the minimal length of a CNV was arbitrary defined at 1 kb. With the advent of next-generation sequencing and new generation arrays, several studies use a minimal length of 500 bp.

**Copy Number Polymorphisms (CNP)**

Common CNVs shared by >1% of a population are referred to as copy number polymorphisms.

**Copy Number Aberration (CNA)**

Copy number aberrations refer to CNVs identified in oncogenomics studies. These aberrations can be germline (predisposition to cancer) or somatic (present in the tumor cell but not in the “normal” diploid cell from the same donor). Somatic copy number aberrations are abbreviated as SCNA. This abbreviation does not suggest whether a given aberration is a driver (initial mutation that led to tumor development and progression) or a passenger event (molecular aberration that is the consequence of one or several driver events).

**Insertion/deletion (indel)**

An indel describes the relative gain or loss of a segment of one or more nucleotides in a genomic sequence.

It is used when the direction of copy number change cannot be defined. For example when it is not clear whether the variant is an insertion in the reference genome or a deletion in the genome of interest. Indels are typically used to denote small-scale variants (smaller than 1 kb in length).

**Segmental duplication (also called low-copy repeat or duplicon)**

A segment of DNA with a length greater than 1 kb that occurs in two or more copies per haploid genome. The different copies share at least 90% of sequence identity. These segments can also be CNVs. Due to the high sequence similarity between the duplicated sequences, segmental duplication predispose to non-allelic homologous recombination.

The observation that CNVs and CNPs (here collectively referred to as CNVs) could occur both in normal (Iafrate et al., 2004; Sharp et al., 2005; Feuk et al., 2006; Fiegler et al., 2006; Freeman et al., 2006; Redon et al., 2006; Kidd et al., 2008, 2010; Perry et al., 2008; Conrad et al., 2010; Valsesia et al., 2012) and disease (Firth et al., 2009; Zhang et al., 2009; Grozeva et al., 2010; Walters et al., 2010; Wellcome Trust Case Control Consortium et al., 2010; Jacquemont et al., 2011) populations has opened a new chapter in human genomics. CNVs have been explored in European (Redon et al., 2006; Li et al., 2009; Gayán et al., 2010; Valsesia et al., 2012), African (Matsuzaki et al., 2009; McElroy et al., 2009), and several Asian populations: Chinese (Lin et al., 2008), Japanese (Takahashi et al., 2008), Korean (Kang et al., 2008; Jeon et al., 2009). Comparisons have been performed between human populations (Jakobsson et al., 2008; Conrad et al., 2010; Kato et al., 2010) and across apes (Nistér et al., 1987; Conrad and Hurles, 2007; Kidd et al., 2008, 2010). CNVs constitute a non-negligible part of the genetic diversity, with consequences in term of evolution and disease susceptibility (Conrad and Hurles, 2007). Consequently, their detection and association with quantitative traits and clinical phenotype constitute an important step toward a better understanding of disease etiology. However, such their detection remains challenging. There are numerous factors in the data generation and computational analyses that can lead to spurious associations. Finally, the sheer amount of data that can be generated already for a single subject imposes severe challenges in terms of data interpretation. In this review, we provide an overview of the different platforms and analytical steps from CNV detection to association with clinical traits. We discuss promising strategies to interpret structural variations in the context of personalized medicine.

## High-Throughput CNV Discovery Platforms

Gross copy number (CN) alterations were initially detected with karyotyping in the early days of cytogenetics. Several large-scale aberrations (Pepler et al., 1968; Dowjat and Wlodarska, 1981; Nistér et al., 1987) were identified before the development of higher resolution techniques. Fluorescence *in situ* hybridization (FISH) has increased this resolution, enabling the detection of *sub-microscopic* CNVs that could not be detected with karyotyping. Today, the most widely used techniques can be classified as amplification-based (polymerase chain reaction), hybridization-based (FISH, comparative genome hybridization, and SNP arrays) or sequencing-based. These techniques differ in precision, throughput, and resolution. In this review we focus on genome-wide CNV discovery platforms: DNA microarrays (CGH and SNP) and NGS.

### Microarray-based methods

#### Single nucleotide polymorphism genotyping arrays

**Figure 1:**
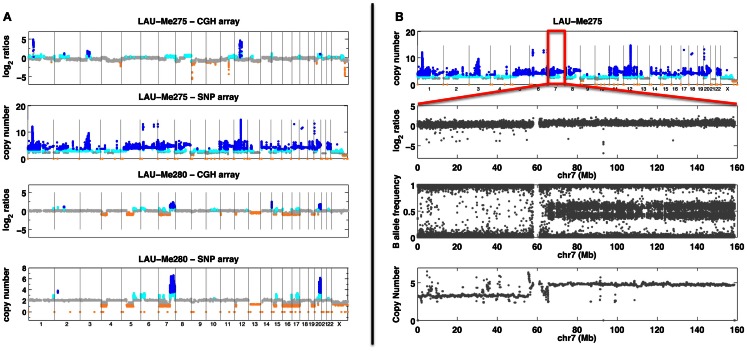
**SNP and CGH array analyses**. **(A)** Analyses with SNP and CGH arrays of two melanoma samples (Me275 a tetraploid sample and Me280 with large deletions). Probe/SNP are plotted as a function of their genomic position on the *X* axis. *Y* axis for CGH arrays corresponds to hybridization ratios. *Y* axis for SNP arrays corresponds to the predicted copy number. Colors indicate a copy number state (orange <2 copies; gray = 2 copies; cyan = 3 copies; dark blue >3 copies). **(B)** Analysis of the Me275 sample with SNP array. The top panel shows genome-wide copy number. Subsequent panels show chromosome 7 with, from top to bottom: hybridization log2 ratio, B allele frequency and copy number prediction.

The Hapmap project (The International HapMap Project, 2003) has played a major role in the discovery and characterization of single nucleotide polymorphisms (SNP). Investigation of genotype data from trios played a major role in the identification of CNVs from SNP genotyping arrays. Indeed CNVs could be detected from the following patterns: (1) SNPs violating Mendelian inheritance principle (Conrad et al., 2006), (2) clusters of genotyping errors, and (3) regions not in Hardy–Weinberg equilibrium (McCarroll et al., 2006). Both McCarroll et al. (2006) and Conrad et al. (2006) showed that these events corresponded to deletions. This prompted the need to re-analyze SNP genotyping arrays for CNVs. Although these arrays were not primarily designed for CNV analysis, it is possible to obtain a CN ratio by combining the intensities of the two alleles and normalizing this quantity with respect to reference. CNV can then be detected by identifying significant deviations from the baseline CN ratio. Some publicly available software combines CN and allelic ratio (the ratio of the allele intensities) to improve CNV detection (Table 1). Such strategy can be applied both for tumor analysis (LaFramboise et al., 2005; Attiyeh et al., 2009) (Figure 1) and diploid sample analysis (Colella et al., 2007; Wang et al., 2007a; Coin et al., 2010). Now, genotyping arrays include both SNP probes and CN probes to cover previously established CN variant regions. The choice of a method will depend on several factors: (1) which platform is to be analyzed (Illumina or Affymetrix), (2) the desired output (discrete or continuous CN prediction), and (3) the type of DNA to be analyzed (germline or somatic CNV analysis). Methods should not be used only with their default parameters. Provided that technical replicates are available, the analyst should compare different methods in combination with different parameters. This can lead to significant improvement both in term of sensitivity and specificity (Valsesia et al., 2011, 2012).

**Table 1:** **Examples of algorithms for the detection of structural variants from array data**.

Software	Affymetrix		Illumina			CGH	Method	Use allelic intensities	Multi-sample analysis	Copy number output	URL
	6.0	500 k	1 M	610 k	550 k	
CRLMM (Scharpf et al., 2011)	X		X	X	X		Corrected robust linear model with maximum likelihood distance	X	X	Allele-specific copy number (CN)	http://www.bioconductor.org/packages/release/bioc/html/crlmm.html
ASCAT (Van Loo et al., 2010)	X	X	X	X	X		Allele-specific piecewise constant fitting	X		Allele-specific copy number (CN)	http://heim.ifi.uio.no/bioinf/projects/ASCAT/
GMM (Valsesia et al., 2012)	X	X	X	X	X	X	Gaussian mixture model		X	Continuous CN	http://www2.unil.ch/cbg/index.php?title=GMM
PICNIC (Greenman et al., 2010)	X						Hidden–Markov model (HMM)	X		Continuous CN + CN genotypes	http://www.sanger.ac.uk/genetics/CGP/software/PICNIC/
GLAD (Hupé et al., 2004)	X	X	X	X	X	X	Adaptive weight smoothing			Discrete CN	http://www.bioconductor.org/packages/release/bioc/html/GLAD.html
PennCNV (Wang et al., 2007a)	X*	X*	X	X	X		HMM	X	Trios only	Discrete CN + CN genotypes	http://www.openbioinformatics.org/penncnv/
Birdsuite (McCarroll et al., 2008)	X						HMM	X	X	Discrete CN + CN genotypes	http://www.broadinstitute.org/scientific-community/science/programs/medical-and-population-genetics/birdsuite/birdsuite
QuantiSNP (Colella et al., 2007)	X*	X*	X	X	X		HMM	X		Discrete CN + CN genotypes	http://www.well.ox.ac.uk/QuantiSNP/
Affymetrix.aroma (Bengtsson et al., 2008)	X	X					Copy number estimation using robust multichip analysis (CRMA)		X	Unclassified segments	http://www.aroma-project.org/
cn.Farms (Clevert et al., 2011)	X	X					Probabilistic latent variable model		X	Unclassified segments	http://www.bioconductor.org/packages/release/bioc/html/cn.farms.html
GADA (Pique-Regi, 2008)	X	X	X	X	X	X	Sparse Bayesian learning		X	Unclassified segments	http://biron.usc.edu/~piquereg/GADA/
CBS (Olshen et al., 2004; Venkatraman and Olshen, 2007)	X	X	X	X	X	X	Binary segmentations assessed by permutations			Unclassified segments	http://www.bioconductor.org/packages/release/bioc/html/DNAcopy.html

X* indicates a software not initially designed for such analysis, but that might be used providing upon additional pre-processing steps.

#### Comparative genome hybridization arrays

Comparative genome hybridization compares the relative CN of a test DNA with respect to a reference DNA (Kallioniemi et al., 1992; Ylstra et al., 2006; Carter, 2007; Redon et al., 2009). The two DNA samples are labeled with different dyes (red or green), and then hybridized competitively. A ratio of relative CN changes can then be measured; significant deviations from the baseline indicate CN gains or losses with respect to the reference genome (Figure 1). Initial CNV detection was made using arrays having a resolution close to 50 kb (Fiegler et al., 2006; Redon et al., 2006). Current CGH arrays, such as Agilent 1 M arrays, have a median resolution of one probe every 2.1 kb. Such resolution is not as good as the one obtained from recent SNP arrays (<500 bp) but the signals obtained from few CGH probes tend to be more reliable than those obtained from few adjacent SNPs (Curtis et al., 2009; Pinto et al., 2011) and although allele-specific CN cannot be inferred from CGH (as opposed to SNP arrays), these arrays remain popular for the detection of CNV both in somatic (tumors) (Kallioniemi et al., 1992; Pinkel and Albertson, 2005; Bignell et al., 2007) and in constitutional diagnostics (Oostlander et al., 2004; Shaffer and Bejjani, 2006; Edelmann and Hirschhorn, 2009; Boone et al., 2010).

#### Sequencing-based methods

Today, NGS technologies allow one to sequence millions of reads in parallel. New methods for structural variant analysis were developed (Medvedev et al., 2009; Dalca and Brudno, 2010; Ruffalo et al., 2011; Koboldt et al., 2012) including paired-end mapping (PEM), read-depth analysis, split-read strategies, and sequence assembly comparisons. References to freely available tools are given in Table 2.

**Table 2:** **Algorithms for the detection of structural variants from NGS data**.

Strategy	Approach	Reference
Paired-end mapping	Detection of discordant end-pairs	Tuzun et al. (2005), Chen et al. (2009), Korbel et al. (2009)
	Clustering of end-pairs	Korbel et al. (2007, 2009), Kidd et al. (2008), Hormozdiari et al. (2009), Lee et al. (2009)
Read-depth analysis	Detection of local change points	Campbell et al. (2008), Chiang et al. (2009), Klambauer et al. (2012)
	Detection of outliers compared to the read-depth baseline	Alkan et al. (2009)
	Event-wise testing	Yoon et al. (2009)
Split-read analysis	Identification of breakpoints with a pattern growth algorithm	Ye et al. (2009)
Sequence assembly analysis	*De novo* assembly and comparison to reference genome	Simpson et al. (2009), Li et al. (2010), Simpson and Durbin (2012)	Burrows–Wheeler transform	Simpson and Durbin (2010, 2012)
	Simultaneously assembly of multiple eukaryotic genomes	Boone et al. (2010), Simpson et al. (2009), Iqbal et al. (2012)
	Detection of small indels through local reassembly	Massouras et al. (2010)
Mixed strategies	Combines both paired-end mapping and read-depth analysis	Medvedev et al. (2010)

#### Paired-end mapping approaches

**Figure 2:**
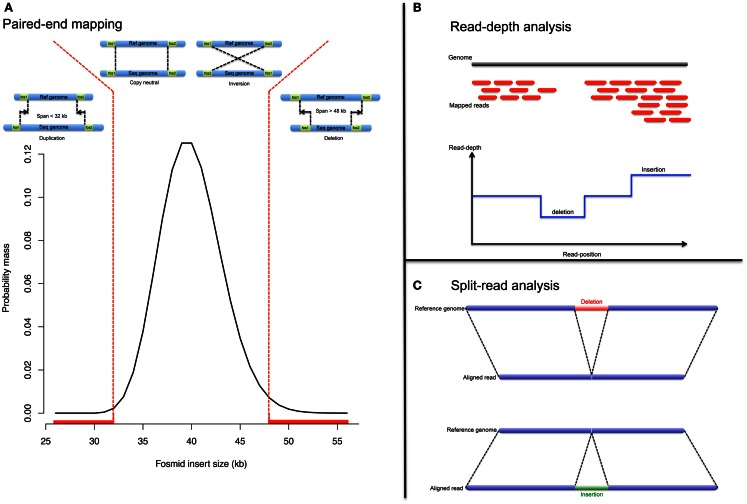
**NGS approaches**. Analytical strategy to detect CNV from NGS data: **(A)** pair-end mapping approached, **(B)** read-depth approach, and **(C)** split-read approach.

Before the advent of NGS, structural variants were detected from fosmid paired-end sequencing (Tuzun et al., 2005; Kidd et al., 2008). The principle is as follow: (1) the genomic sequence is fragmented and cloned into fosmids. (2) Ends of the cloned fragments are sequenced using universal primers and aligned to the reference genome. (3) Paired-ends, discordant in length or direction, indicate respectively possible indels or inversion (Figure 2A). PEM enables precise breakpoint determination and performs well even in the presence of repetitive elements (LINE, SINE). However it fails when both paired-ends map within repeats. Also the detection resolution is limited to the distance between pairs; therefore, neither large nor very small rearrangements can be detected, with the exception of large deletions.

#### Read-depth approach

The read-depth analysis investigates change in read coverage compared to an expected depth distribution (Figure 2B). Mutual information about paired-reads is used to improve the mapping quality and to detect complex and large rearrangements. However read-depth analysis is challenging in repeat-rich regions (due to mapping issues).

#### Split-read approach

The split-read strategy entails in gapped-alignment of reads onto candidate breakpoints (Figure 2C). The strategy is to detect paired-reads where only one end is uniquely mapped onto a reference genome (Ye et al., 2009). The assumption is that the second paired-read could not be mapped, even with few mismatches allowed, because it corresponds to a deletion or insertion breakpoint. The mapped-read is used as an anchor and knowing both a maximum event length and the direction to search for the unmapped-read; alignment of the unmapped-read can be performed either by splitting it into two or three fragments whereby the former indicates a deletion event and the latter indicates an insertion event (Figure 2C).

#### Sequence assembly comparison

Provided a high sequencing depth, *de novo* assembly can be attempted (Simpson et al., 2009; Li et al., 2010; Iqbal et al., 2012; Simpson and Durbin, 2012) such that a sequence comparison can be made with the reference genome to identify deletions and insertions. The advantage of *de novo* assembling over PEM approaches is that deletions or insertions smaller than the paired-end insert size can be detected. But on the other hand, *de novo* assembling is very difficult for repeat-rich regions and until recently (Iqbal et al., 2012) was only possible with high read-depth. When this criterion is not met, several experiments can be pooled together (The 1000 Genomes Project Consortium, 2010).

The above techniques present different and complementary advantages. Combining several approaches definitely empowers the detection of structural variations (Mills et al., 2011).

### Pitfalls in CNV analyses

#### The need for adequate design and laboratory quality control

Despite tremendous improvement in the different technologies and analytical methods, CNV detection remains a difficult task (Wineinger et al., 2008; Curtis et al., 2009; Winchester et al., 2009; Eckel-Passow et al., 2011; Haraksingh et al., 2011; Pinto et al., 2011; Valsesia et al., 2011, 2012). Both DNA microarrays and NGS are prone to batch effects. Date of experiment, plate id, experimenter or ozone levels are all factors that can influence CNV prediction. Batch effects can have very severe consequences and lead to spurious associations. Inappropriate sample randomization, such as genotyping cases and controls within separate batches, is the worst-case scenario in case-control studies. Unfortunately such a scenario is all too common and is typically discovered late in the data generation process. Therefore careful experimental planning and quality control, including thorough investigation about putative batch effects, should be considered as part of the core analysis.

A number of approaches should be considered such as (1) detecting outliers at different laboratory QC steps, (2) using positive and negative controls to check the consistency between batches, (3) performing principal component analyses or other multivariate analyses to detect possible batch effects, (4) Using technical replicates to check consistency of the results and estimate noise levels in the data. In addition, to these common pitfalls in any CNV analysis, there are other limitations that are inherent to either DNA microarrays or NGS experiments.

#### DNA microarray limitations

DNA microarrays suffer from several limitations, notably the measured CN ratio derived from fluorescence intensities is very noisy and is subject to artifacts such GC-biases, probe spatial auto-correlation, non-specific hybridization, differences between color dyes for CGH arrays, and allelic crosstalk for SNP arrays. Numerous normalization procedures have been proposed (Marioni et al., 2007; Bengtsson et al., 2008; Chen et al., 2008; Diskin et al., 2008; Fitzgerald et al., 2011) to address these issues. Nevertheless these normalizations, e.g., LOESS smoothing, can mask small CN changes and often are not sufficient to avoid false-positives. Typically, a number of adjacent probes will be required to define a CNV but *de facto* this prevents the detection of very small CNVs.

Also repeat-rich regions and regions close to segmental duplications remain poorly covered, owing to the challenge at designing probes with limited risk of cross-hybridization. These genomic regions are highly dynamic (prone to rearrangements) and may thus be enriched for CNVs. To overcome this density limitation, the latest SNP array generation combines both SNPs and *non-polymorphic* probes to cover CNV regions (McCarroll et al., 2008).

Finally, DNA microarrays do not provide a CN digital readout due to hybridization saturation. Several methods (Greenman et al., 2010; Van Loo et al., 2010; Scharpf et al., 2011) for SNP arrays allow a continuous CN prediction that is not limited to a discrete five-state classification (CN = 0, 1, 2, 3, or >3). Although precise CN estimation remains difficult (for example to distinguish between six and seven copies), such estimates are sufficient to identify loci to be re-assessed with targeted methods. Continuous CN prediction is possible due to the use of allele-specific information (allelic intensity ratios). Traditional CGH arrays do not include such information, but newer arrays developed for diagnostic purpose combine both CGH and SNP probes resulting in a better CN classification and allowing the detection of uniparental dysomy and copy-neutral LOH.

#### NGS limitations

Next-generation sequencing offers several advantages over DNA arrays in particular; it allows detection of very small variants (indels, SNPs) and inversion. It can estimate exact breakpoint location and does not suffer from hybridization saturation allowing a better (digital) estimation of high CNs. However CNV analysis from NGS data is not trivial (The 1000 Genomes Project Consortium, 2010; Mills et al., 2011). Biases can be introduced by the experimental protocol and need to be addressed. Sequence capture arrays, used for exome sequencing, tend to introduce biases due to the range of GC content that is captured (hybridized) (Dohm et al., 2008; Klambauer et al., 2012; Li et al., 2012). Sequence read quality score might be biased due to the presence of indels/CNVs, these scores need to be re-calibrated with local realignment around known indel sites (McKenna et al., 2010; DePristo et al., 2011). In addition, the coverage will not be uniform across the genome: longer genes will have in average a better coverage compared to smaller ones; and low-complexity regions will have low coverage. Thus modeling of read-depth across samples at each position and across samples helps to account for such biases, to estimate the noise, and to control the false discovery rate (FDR) by filtering noisy predictions (Klambauer et al., 2012). Another promising approach is to use singular value decomposition to detect rare CNVs and to infer CNP genotypes from exome sequencing data (Krumm et al., 2012). The NGS field is still evolving and more sophisticated methods are frequently made available (Table 2). A promising strategy to limit the risk of false positives, in particular in the context of clinical diagnosis, is to predict CNVs using multiple algorithms (The 1000 Genomes Project Consortium, 2010; Sudmant et al., 2010) and/or using methods that allow FDR control (Klambauer et al., 2012).

#### Post-filtering and post-processing steps

**Figure 3:**
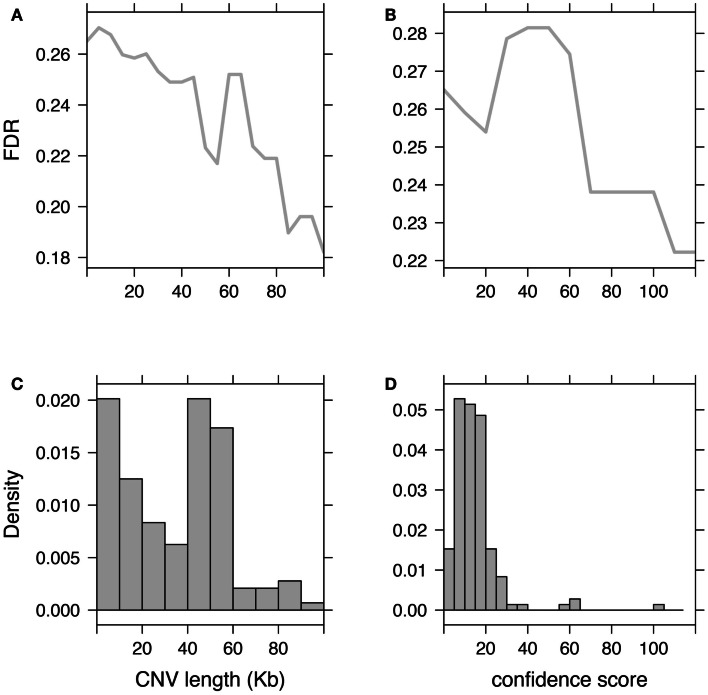
**Impact of CNV post-filtering on false-discovery rate (FDR)**. Illustration of the FDR evolution when discarding CNVs based on their length **(A)** or based on their confidence scores **(B)**. **(C,D)** Show respectively histograms of CNV length and CNV confidence score. Fluctuations in these histograms (such as inversion of the proportion “small CNVs over long CNVs” or “low-confidence over high-confidence CNVs”) are associated with non-monotonic changes in the FDR curve.

Subsequently to CNV detection, additional filtering and processing are often needed to discard possible false-positives. These steps, referred to as either post-filtering or post-processing, are essential prior to any attempt to associate CNVs with clinical/phenotypic traits because false-positives are likely to create spurious associations. Moreover, we showed that a high FDR decreases significantly the discovery power of omics studies (Clevert et al., in press). These post-filtering steps aim at removing either dubious samples or probes. Subjects predicted with too many CNVs as compared to other subjects from the study, should be discarded. An aberrant number of CNVs has proved to be a proxy for poor data quality and/or high FDR. Probe filtering can involve discarding CNV regions that are too rare in the population (for example seen in less than three individuals). But this might remove putative rare CNVs, which are of most importance for association studies and contrary to some common CNVs may not be tagged by SNPs (Redon et al., 2006; Stranger et al., 2007; Conrad et al., 2010; Wellcome Trust Case Control Consortium et al., 2010). These filters remain useful as they discard many false positives and in the context of association studies decrease the multiple-testing burdens. Alternative filtering criteria may flag or use models that account for CNVs with low-confidence score or that are too short to support the call (Figure 3).

## CNV Genome-Wide Association Tests

### General CNV-GWA framework

**Figure 4:**
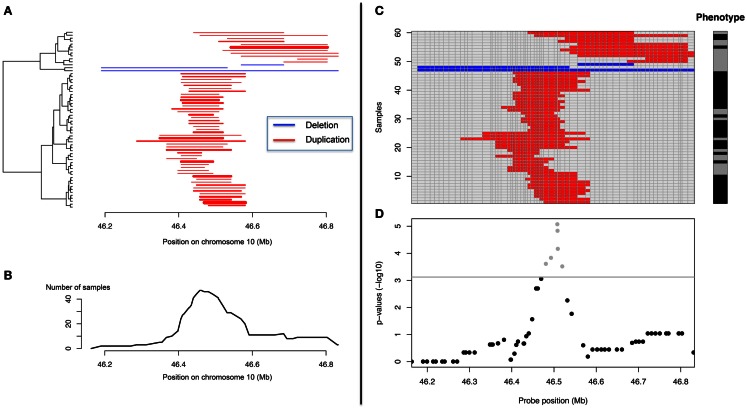
**Representation of CNV data and CNV-GWA analysis**. **(A)** CNV representation on chromosome 10 (*X* axis) for different subjects (*Y* axis). **(B)** Frequency representation of the same CNV. **(C)** Matrix-based representation of the CNV along with the phenotype of the different subjects. **(D)** Representation of the CNV association results.

Association between a given trait and a CNV locus can be performed in several ways. For quantitative traits linear regressions are very popular while logistic regression, Fisher’s exact test or Armitage–Cochrane trend test are often used with binary traits. All these tests may apply at single probe level, but not for CN regions. CNVs across subjects do not necessarily have the same boundaries (Figure 4) and defining a “consensus” CNV locus is not trivial. This problem is frequently ignored and association tests are made using probe-level CN information (Figure 4). Such an approach, assumes that all samples were assayed on the same platform and that data can be combined into a matrix samples by probes, where each element corresponds to a predicted CN. Then association tests can be performed independently for each probe. Since adjacent probes may carry the same information, many tests are redundant. This might not be a computational issue; however it is problematic in terms of multiple-testing corrections. A number of procedures have been previously proposed to identify the number of independent tests in SNP-based genome-wide association tests (GWAs) and would prove useful with CNV-based GWAs (Cheverud, 2001; Nyholt, 2004; Gao et al., 2008).

“Aligning CNVs” from different subjects and identifying the consensus CNV can be useful to identify clusters of CNVs with similar boundaries and help interpretation (Figure 4). This can be done with the so-called merge-by-overlap approach (Conrad et al., 2006; Redon et al., 2006), where CNVs from different individuals are merged into the same CNV region if their reciprocal overlap satisfies a minimal cut-off [>50% is frequently used (Conrad et al., 2006; Redon et al., 2006)]. We proposed recently another approach based on principal component analysis and clustering (Valsesia et al., 2012). Once “aligned,” a matrix CNV by subjects can be derived and the association tests can be performed as aforementioned.

### Differences between genome-wide CNV analysis and genome-wide SNP analysis

Conducting a genome-wide CNV analysis differs greatly from conducting a genome-wide SNP analysis. CNVs and SNPs can both be mined from SNP genotyping arrays, yet data needed for their detection are different. SNP genotypes can be predicted from the two measured allelic intensities while CNVs can be predicted by combining several type of information such as CN ratios and allelic intensity ratios. Methods like Birdsuite (McCarroll et al., 2008) can also integrate SNP genotype data and use prior information such as regions of known CNVs to improve the CNV detection.

Another difference is that SNP analysis is carried out using the whole cohort, while CNV analysis can be performed using either the whole cohort (multi-sample analysis) or sample-wise (each sample is analyzed independently from the others). While SNP genotyping is a fairly standardized procedure; CNV genotyping remains challenging and is prone to high false-positive rates. Therefore, while SNP genotypes can be obtained with a very high prediction confidence; CNV predictions have higher uncertainty levels. These uncertainty levels greatly challenge the subsequent CNV association with a given phenotype or clinical trait.

In addition, these two types of analyses differ in the number of independent tests that are performed. This difference has consequences in the correction for multiple testing. While for SNPs the ratio between the number of tested SNPs and the effective number of truly independent tests is ∼2.5-fold (Han et al., 2009) (in the case of HapMap SNPs), for CNV probes this ratio is several folds higher. We showed recently with the Colaus cohort (Valsesia et al., 2012) (a population-based health survey with more than 5,600 subjects genotyped on Affymetrix 500 k SNP arrays) that CN predictions obtained at 490 k autosomal SNPs could be compressed into about 8 k distinct CNV regions, including both rare and common CNVs. This number of regions gives a first approximation about the number of independent tests. Using the simple ℳ method (Gao et al., 2008), we estimated that the number of truly independent tests was 6,643 corresponding to a 74-fold difference compared to the probe-level CN predictions. Therefore, while for SNP analysis the difference between number of SNPs and number of independent tests is negligible, this quantity is much greater for CNVs and can cause substantial *p*-value deflation, as can be observed with QQ-plots.

For these reasons, a genome-wide CNV analysis, such as a CNV-GWA, is often considered as a secondary analysis, after an initial SNP-GWA. Studies, like those of the GIANT consortium, often check whether SNPs discovered to be associated with a certain trait could potentially tag underlying CNV associations. Two BMI associations (Willer, 2009; Speliotes et al., 2010) (near the *NEGR1* and *GPRC5B* genes) have been identified as potentially driven by deletions.

### Frequent issues in CNV-GWAs

Copy number variations genome-wide associations (GWAs) are much more challenging than SNP-based GWAs, mostly because of the uncertainty of the predicted CNVs. This may explain the lack of published reports from CNV-GWAs. This uncertainty in CN can be tackled by missing data likelihood methods resulting in the usual test statistics (likelihood ratio, Wald test). However these methods can be computationally intensive and the speed of convergence (as sample size tends to infinity) ensured by the central limit theorem is not always as fast as it is for normal linear models.

**Figure 5:**
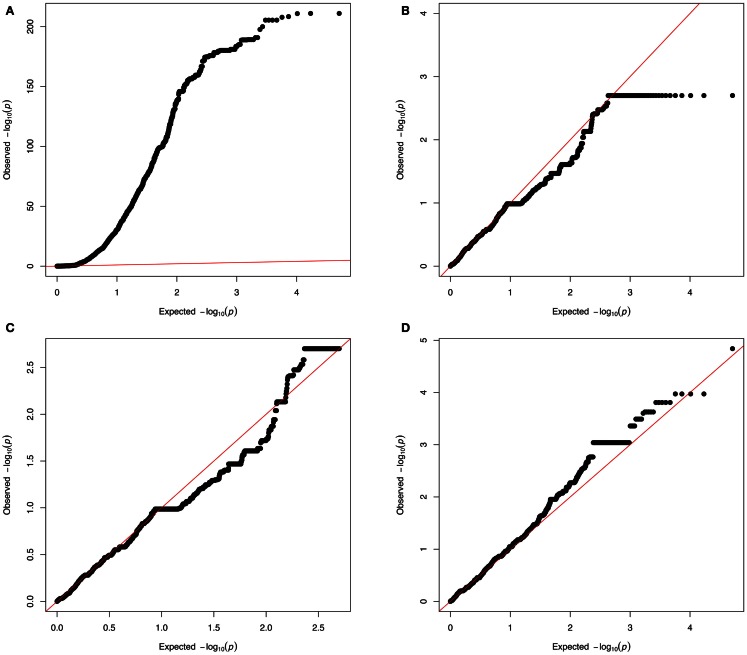
**QQ-plots investigation**. From a real dataset: copy number predictions for more than 3,600 individuals at 95,770 probes from chromosome 1; association was tested with either a simulated phenotype **(A–C)** or a real phenotype **(D**). The simulated phenotype corresponds to normally distributed data influenced by a confounding factor [here the first principal component (PC1) obtained from the matrix of copy number predictions]. **(A)** Shows a strong *p*-value inflation (lambda∼65) that is due to the confounding factor (PC1). **(B)** Corresponds to results from a model where PC1 is added as a covariate (to adjust for the confounding effect). Yet **(B)** shows a slight *p*-value deflation (lambda ∼0.87). This deflation is due to the fact that the tested probes are assumed to be independent while many of these probes correspond to a same CNV region (thus the presented *p*-values are not from truly independent tests). **(C)** Shows a QQ plot adjusting for PC1 and where *P*_0_ (the *X* axis) accounts for the fact that probes can come from the same CNV region. Such plot can be done (in the R programing language) by setting the vector of expected *p*-value (*X* axis) as *P*_0_ < −seq[1/*N*,1,by = (1 − 1/*N*)/(*n* − 1)] where *N* is the number of CNV regions (number of effective tests) and *n* is the total number of CNV probes (number of observations). **(D)** Shows results from association with real data (here body mass index). In these QQ-plots, points with identical *p*-values correspond to rare, but rather long CNVs that produce multiple identical probes.

Non-Gaussian test statistic distributions can lead to spurious associations (Kutalik et al., 2011) and give rise to inflated *p*-values (as can be detected with QQ-plots, see Figure 5A). Although genomic control methods (Devlin and Roeder, 1999) allow correcting for inflated *p*-values in most cases, critical assessment of the CNV pipeline remains necessary both for sensitivity and specificity. Combining methods that estimate FDRs (Clevert et al., 2011; Klambauer et al., 2012) with technical replicates is essential to achieve a good sensitivity-specificity compromise. Figure 5D shows a QQ plot where neither strong *p*-values inflation nor deflation can be seen.

Inflated *p*-values (Figure 5A) can be due to various violations of the model assumptions, e.g., non-normal trait distribution, dependence between tests, or confounding effects such as population stratification (including population admixture), cryptic- or familial-relatedness. Careful covariate selection and diagnostic plots are needed to address the two first issues. For admixture and population stratification, many methods have been proposed to detect and adjust them (Cardon and Palmer, 2003; Rosenberg et al., 2010).

Copy number variations-GWAs can also produce deflated QQ-plots (Figure 5B) owing to the fact the number of tested markers is much greater than the number of truly independent tests. Methods used in multiple-testing adjustment in SNP-GWAs (Cheverud, 2001; Nyholt, 2004; Gao et al., 2008) can be useful to identify CN markers corresponding to independent tests and to produce the corresponding QQ plot using those markers only. QQ-plots can also be produced so that the expected *p*-value vector (P0) reflects the fact that the number of probes (*n*) corresponds to a smaller number of CNV regions (*N*) (see Figure 5C).

Controlling for false positives may in some cases require investigating subject-level data (profile of CN ratio and profile of allelic ratio), CNV frequencies, and the genomic distance between the different signals. Correlated signals from probes adjacent to each other’s would indicate a partially detected CNV (i.e., disrupted CNV prediction) while isolated signals located on different chromosome would more likely correspond to spurious associations. Increasing the stringency filter on very rare CNVs (e.g., removing CNVs with frequency smaller than 1/1000) might avoid the latter issue.

### Analysis of common and rare CNVs

Distinction should be made between analyzing common and rare CNVs. Common CNV shared by >1% of the population are referred to as CNPs. CNPs correspond mostly to ancestral events and segregate in the population with different allele frequencies [owing to the fact that many are multi-allelic (Redon et al., 2006; McCarroll et al., 2008)]. Studies from the WTCCC (Wellcome Trust Case Control Consortium et al., 2010) found that only very few CNPs were likely to be associated with common diseases. It is likely that the effect size of CNPs is modest, and that lack of standardization between studies and small-sample size challenge the identification of association signal. Instead of discrete (continuous), CN genotypes are preferred to be tested (McCarroll, 2008). A number of software (Wang et al., 2007a; McCarroll et al., 2008; Greenman et al., 2010; Van Loo et al., 2010) packages exist to compute CN genotypes rendering such analyses possible.

For rare CNV association studies, a large sample size is needed to obtain the required statistical power. This can be achieved by pooling data from different cohorts (Walters et al., 2010; Jacquemont et al., 2011). This task is challenging due to the differences between cohorts, platform vendors (and thus genomic content), analytical methods and even FDR. Re-analysis of these cohorts genotyped on more homogeneous platforms would enable rare CNV-GWAs possible (Voight et al., 2012). Also, other Illumina chips share the vast majority of the Illumina370 probe set, which can be a common set of probes to use. Meta-analysis of case-control associations can be extended to rare variants. For binary traits, collecting case and control counts for a given CNV facilitates efficient meta-analysis. For continuous traits, however, inverse-variance weighting meta-analysis may be sensitive to slight deviations from normality of the test statistics, thus requiring robust extensions.

### Toward better methods for CNV-GWAs

Most of the association tests rely on discrete CN classification (hard-classification). Given the CN prediction uncertainty and the important false-positive rate, hard-classification is no longer sufficient (Barnes et al., 2008). We showed previously that for SNP-based GWAs, modeling genotype uncertainty was significantly better than using called genotypes when data were of low quality (Kutalik et al., 2011). Specific strategies have been proposed for CNV-GWAs: the case-control framework from Barnes et al. (2008) that applies likelihood ratio testing of CN ratio in cases and controls; the modeling of CN state probabilities in logistic regression (Xu et al., 2011) and methods that can test the CN ratio from family-based design (Ionita-Laza et al., 2008; Murphy et al., 2010).

Since CNVs segregate at different frequencies in different ancestral populations (Jakobsson et al., 2008), recent improvements in SNP-GWAs (Kang et al., 2010) accounting for population structure via mixed-models could be readily extended to CNV-GWAs. Burden tests designed for SNVs (Yang et al., 2008; Asimit and Zeggini, 2010; Neale et al., 2011; Asimit et al., 2012; Kinnamon et al., 2012; Lee et al., 2012a,b; Chen et al., 2013) could also be adopted to combine rare aberrant CN events in a region.

## CNV and Biological/Clinical Interpretation

**Figure 6:**
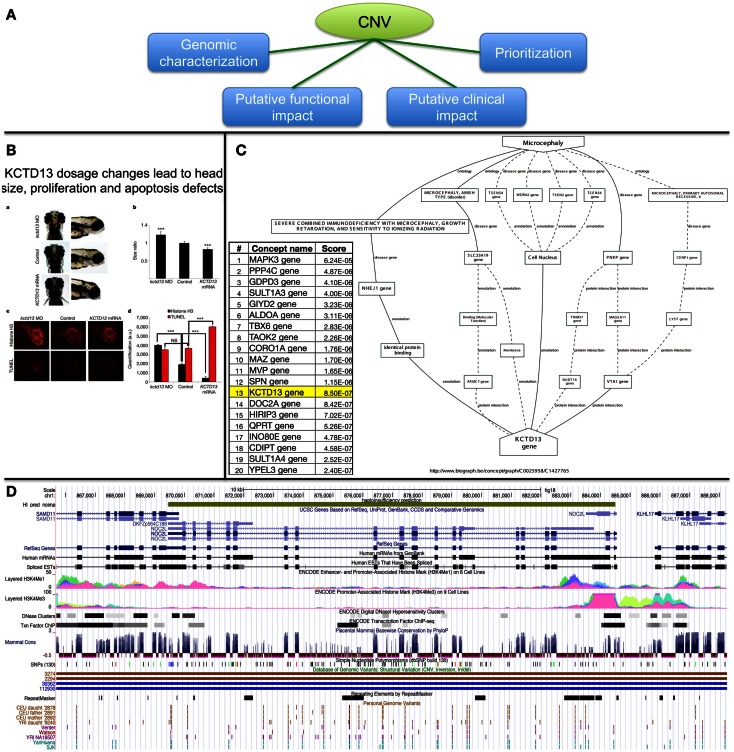
**Possible strategies for CNV prioritization**. **(A)** Overview of possible strategies. **(B)** Functional investigation in animal models (functional impact assessment). **(C)** Genes ranking based on text-mining approaches (prioritization). **(D)** Visualization in genome browser (genomic characterization).

The importance of rare CNVs emerged with a few GWAs (Glessner et al., 2010; Grozeva et al., 2010; Prakash et al., 2010) and many candidate studies (de Cid et al., 2009; Bochukova et al., 2010; Walters et al., 2010; Williams et al., 2010; Jacquemont et al., 2011; Pagnamenta et al., 2011). To date, more than 291,801 CNV regions [from 53 studies, see release dated as November 23, 2012 from the DGV database (Iafrate et al., 2004)] have been identified in the general population and CNVs linked with 65 genomic syndromes are described in DECIPHER (Firth et al., 2009) for more than 7600 patients. With the advent of NGS projects aiming at clinical diagnosis (Vasta et al., 2009; Lupski et al., 2010; Bainbridge et al., 2011; Bamshad et al., 2011; Isidor et al., 2011; Calvo et al., 2012; Haack et al., 2012; Köser et al., 2012; Neveling et al., 2012), thousands of variants can be expected per patient. This poses many problems to clinical labs on how to filter, prioritize, and interpret variants that might potentially be associated with disease susceptibility, progression, and possibly response to treatment. Figure 6A summarizes possible strategies that we discuss below.

### CNV genomic characterization

The first step to understand the potential impact of a single CNV is to investigate its genomic context. For e.g., if the CNV is located within/near a gene, the gene annotation may already provide valuable information (Figure 6D). Vicinity of repeats [including segmental duplications and L1 retrotransposon (Zhang et al., 2009)] as well as specific non-B DNA conformation (Bacolla and Wells, 2004) can be indicative about a genesis mechanism. Presence of miRNA coding sequences, DNase hypersensitive clusters and ChIP-seq binding sites can be clues about possible transcription regulation. Overlap with previously reported hits from SNP-GWAs can also help to pinpoint a particular gene or biological process. A number of tools allow sequence-based annotation and to visualize large amounts of data (Fiume et al., 2012; Flicek et al., 2012; Kuhn et al., 2013). Genome browsers of numerous large-scale datasets such as those from the ENCODE project (ENCODE Project Consortium et al., 2012) proved to be a great asset for CNV annotation, in particular to offer regulatory evidence and facilitate explanation regarding the putative CNV impact in a large range of tissues. These tools and datasets are now widely used by biologists and clinicians to annotate and prioritize their variants. A recent and noticeable addition is the variant effect predictor (McLaren et al., 2010) (VEP, formerly known as the SNP effect predictor). This tool allows annotating SNP, indels, and CNVs from any species using highly curated data from Ensembl (Flicek et al., 2012). VEP can be used directly from within the Ensembl genome browser (usage limited to 750 variants), or remotely using the Ensembl API, or even locally using a stand-alone script (no limitation on the number of variants to be analyzed). Documentation and source code can be retrieved from Variant Effect Predictor^1^. Currently VEP provides indication about the possible consequences as described by the Sequence Ontology (Eilbeck et al., 2005); checks for overlap with known regulatory features and whether the variant falls in a high information part of a transcription factor binding site; check for previously reported variant at the same location and report frequencies from the 1000 Genomes project for known variant. For SNPs, VEP also provides allele/genotype frequencies, a list of tagged variants (as well as LD calculation) and predictions from SIFT (Kumar et al., 2009) and Polyphen (Adzhubei et al., 2010). Future development of VEP will annotate variants with data from animal studies, human ClinVar^2^, Orphanet^3^, LSDBs (HGVS LSDBs Listing)^4^, and summary-level data from DECIPHER v5^5^, UK10K^6^, and EGA (The European Genome-phenome Archive)^7^.

### Investigating the putative functional impact

Assessing the functional impact of CNVs can be achieved by assessing protein levels or kinase phosphorylation status to determine whether transduction signal in a disease-relevant pathways might potentially be affected by the variant of interest (Dos Santos et al., 2004); up to “engineering” the DNA variation in model organisms and study the impact on development. This latter strategy was successfully applied in our quest of candidate genes associated with microcephaly (Jacquemont et al., 2011) (Figure 6B).

Although such experimental analyses are best to dissect the molecular mechanisms and consequences induced by genomic variants; these analyses are challenging and not adapted for large number of candidates. Since CNV can affect gene expression levels (Stranger et al., 2007; Dimas et al., 2009; Henrichsen et al., 2009) assessing whether a list of candidates can potentially induce differential expression (ideally in the same patients) can help with investigating putative CNV downstream consequences. Assessing gene expression levels for a subset of the cohort (with microarrays, targeted approaches, or even RNA-seq) is currently possible with relatively affordable costs for any large-scale genetic study. A caveat to these expression analyses is that the appropriate target tissue is not always available. Most frequently, such analyses are performed on RNA derived from blood cells; e.g., immortalized lymphoblastoid cell lines. Although it can be a good starting point before further investigation, in the foreseeable future using iPS-derived specialized cells would provide better insights.

### Investigating the putative clinical impact

Assessing the clinical impact of a genetic variant is definitely not a trivial task: it requires carefully designed studies and is generally outside of the scope of the initial study that has identified the variant of interest. This section discusses available resources that could help building *a priori* knowledge about the putative impact of a CNV before designing subsequent studies.

Family studies can bring some evidence in support of an association between a CNV and a phenotype. Genetic diagnostic labs routinely use such strategies but the interpretation of these segregation analyses are often hampered by partial penetrance of the CNV under investigation. For instance, a CNV may have been inherited from an unaffected parent and yet be a major factor contributing to the trait in the child (Girirajan et al., 2012). To help address this issue, in depth clinical phenotyping of the patients (and their relatives) as well as sharing clinical case between diagnostic labs are helpful. But ultimately, additional case-control studies are needed.

Today, CNVs identified by clinical labs can be shared through the DECIPHER interface (Firth et al., 2009). DECIPHER is an online repository of CNV and phenotype data whose goal is to enable the clinical interpretation of CN variation (Corpas et al., 2012). The web interface includes a number of tracks (associated syndrome, CNV consensus track, haplo-insufficiency track) that facilitate data interpretation. Other databases have collected CNVs from publications. Although these databases are good resources, they should be used with great caution in the clinical setting (Duclos et al., 2011) mostly because within these databases, CNVs were detected in populations whose participants were not necessarily ascertained clinically and because the CNV frequencies from these studies are not comparable due to differences in design, platform, analytical pipeline, and false-discovery rate.

### Prioritization of many candidate CNVs

The above approaches are useful when a limited number of candidates are to be investigated. To date, software such as Cartagenia are efficient at prioritizing large CNVs (>200 kb) related to diagnosing developmental delay in the clinic.

In the research context, when the number of CNVs is much larger, *in silico* methods are needed to prioritize and filter the calls. Although there is globally a lack of prioritization methods, a number of existing approaches, used in gene expression and SNP-GWAs can be useful. These approaches include text-mining approaches, geneset enrichment analyses, and network-guided analyses.

#### Text-mining approaches

Text-mining is a powerful way to mine the scientific literature and identify links between a concept term (such as a disease name or a MeSH term) and a given gene (Rebholz-Schuhmann et al., 2012). A number of tools already exist (Tranchevent et al., 2008, 2011; Liekens et al., 2011) and are useful to rank a list of genes in the vicinity of candidate CNVs or simply to identify new concepts/genes that link a gene of interest to a disease (Figure 6C). An inherent limitation is that genes that have been extensively studied can influence the ranking. Depending on the statistical framework of the method, genes listed in many publications might be better ranked than genes described with fewer reports. Figure 6C shows that although *KCTD13* was involved in microcephaly in zebrafish, it only ranked 13th out of the 29 genes involved in the 16p11.2 CNV while the *MAPK3* gene ranked first. Nevertheless using multiple algorithms/ontologies (Malik et al., 2006; Yu et al., 2008) and/or using a training set of genes for a biological process of interest (Tranchevent et al., 2008) are simple ways to improve the prediction performance.

#### Geneset enrichment analyses

Geneset enrichment analyses are very popular in gene expression studies and test the overlap with a given biological annotation (molecular pathway, ontology). Several resources are available such as DAVID (Huang et al., 2009), GSEA (Subramanian et al., 2005), and GOstat (Beissbarth and Speed, 2004). These methods have a number of caveats (Pavlidis et al., 2012; Tamayo et al., 2012) and the results require critical interpretation. Therefore combining several recent methods (Richards et al., 2010; Geistlinger et al., 2011) as well as thorough (expert) biological interpretation (to check consistency and relevance of the final annotation) is needed to avoid story-telling (Pavlidis et al., 2012).

#### Network-based analyses

A number of studies (Cancer Genome Atlas Research Network, 2008; Berger et al., 2010; Cerami et al., 2010; Lango Allen et al., 2010; Millstein et al., 2011; Valsesia et al., 2011; Lee et al., 2012c) have been successful by integrating both genomic variants and gene expression, into networks of protein–protein interactions and by identifying sub-networks made of proteins significantly connected to each other, and corresponding to genes/transcripts affected with structural variations and/or differential gene expressions. Such clustering analyses allow restricting a list of candidate genes to those whose products are known (or predicted) to interact with each other, thereby enriching for genes potentially participating to a same biological process.

Furthermore these network-guided analyses allow flexibility in that genes apparently “unaffected” in the dataset but significantly linking other “affected genes” can be identified. Indeed this strategy was successfully applied to glioblastoma (GBM) (Cerami et al., 2010) and identified relevant candidate genes linking known GBM’s genes.

Today, researchers can construct their own network of interactions from gene expression data and text-mining approaches. Such networks are referred as prior knowledge networks (PKNs). Using disease-relevant PKNs (from focused literature and/or relevant gene expression datasets) provides a powerful strategy to connect genes affected by CNVs. Many methods have been proposed to identify SNPs associated with clinical trait using network-guided analyses (Wang et al., 2007b; Raychaudhuri et al., 2009; Lango Allen et al., 2010; Kasarskis et al., 2011; Lee et al., 2011; Millstein et al., 2011; Rossin et al., 2011; Glaab et al., 2012). In fact, these methods are often used in SNP-GWAs and in drug discovery projects. Applying those methods on CNVs and in combination with relevant PKNs is very appealing for the detection and clinical interpretation of CNV sub-networks.

## Discussion

Numerous studies have documented CNVs in a genome-wide fashion and their impact on disease and evolution is clearly established. Yet the detection of CNVs and subsequent association with clinical and functional phenotypes remains very challenging.

Remarkable improvements have been made to call CNVs from recent platforms, yet older generation arrays have not been mined extensively due to a lack of standards (Valsesia et al., 2012). Today, tremendous efforts are invested in NGS projects. Although methods to detect indels and CNVs are still being developed, thousands of structural variants are expected for a single individual. The lack of gold standard, the heterogeneity across platforms and methods, as well as the massive amount of data generated constitute a great challenge for result interpretations. These issues have been known for several years (Pinto et al., 2011), yet the CNV community has not agreed on any standards. Such standards could potentially be set by large genomic projects like the 1000 Genome project (The 1000 Genomes Project Consortium, 2012) or large biomedical projects like DDD (Firth et al., 2011) (deciphering developmental disorders), a DECIPHER initiative.

The largest study to date has revealed very few examples of associations between common CNVs (CNPs) and common disease (Wellcome Trust Case Control Consortium et al., 2010). Moreover, all of the CNPs involved in these associations are well tagged by SNPs. Association between rare CNVs and common/complex disease has been demonstrated with several candidate approaches (McCarthy et al., 2009; Walters et al., 2010; Jacquemont et al., 2011) and several large CNVs (>100 Kb) from genome-wide analyses have been found associated with schizophrenia as well as other neuro-developmental disorders (International Schizophrenia Consortium, 2008; Stefansson et al., 2008; Walsh et al., 2008; Xu et al., 2008; Kirov et al., 2009; Williams et al., 2010; Cooper et al., 2011; Grozeva et al., 2012; Malhotra and Sebat, 2012). Yet the literature remains sparse regarding successful genome-wide investigations for other traits/diseases or regarding smaller CNVs. This highlights the need (1) for new methods for CNV-GWAs, (2) to re-investigate study design with family-based design instead of case-control design with unrelated controls (from the general population), and (3) for thorough clinical phenotyping.

Many visualization platforms and analytical methods are available for understanding the impact of (coding) SNPs and somatic mutations. Yet little (almost nothing) is available for clinical interpretation of indels and CNVs. Presently a few companies develop and sell software to research and clinical labs. Beside the cost of these tools, these are often regarded as *black boxes*. The underlying algorithms and code are not made available thus the user cannot check whether state-of-art methods are used and cannot understand in finer details how the result was obtained. The functionalities are often limited to data management and visualization. Only a few basic analyses are provided for clinical interpretation and there is very little flexibility to expand the existing functionalities or even to integrate new ones. In this review, we have highlighted a number of strategies for CNV clinical interpretation. Although those methodologies are not necessarily available within a single software, there are numerous individual and freely available tools that can be used.

With the rapid evolution of the different platforms and analytical methods there are knowledge gaps to be filled. These gaps can range from the appropriate design of a large-scale genetic study, to the different steps from data generation to computational analyses, results validation, and interpretation. Today, there is a need for computer-literate biologists and clinicians, as well as bioinformaticians embedded within wet-labs and clinical diagnostic labs. To improve the communication between the different actors, there is a strong need for developing cross-competencies and to use a common vocabulary. Most clinicians have access to continuous education; similarly biologists and bioinformaticians can benefit from various university formations/seminars. Continuing these efforts is worthwhile and additional formations focused onto the interpretation of omics-data in a clinical setting are needed. These synergies and complementarities between the different parties, as well as a shared common knowledge are critical components to progress toward a better data interpretation and hopefully toward personalized medicine.

Finally, extensive and accurate phenotyping, as well as data sharing using centralized and secure databases like DECIPHER, are essential to speed-up the CNV clinical interpretation and to bridge between research and diagnostic labs.

## Perspectives

Today the pathogenic contribution of CNVs to rare inherited diseases is well established, yet the contribution to complex traits remains unclear. In addition, most genotyping assays rely on markers that do not violate Mendelian inheritance principles and that are in good Hardy–Weinberg equilibrium in the general population (HapMap). This excludes genomic regions that are highly dynamic (like segmental duplications or low-complexity regions) and that are subject to recurrent CN changes. With the recent improvements in the NGS field (longer reads, higher sequencing depth, newer mapping methods), analysis of these regions becomes possible (although very challenging). Careful investigation of these regions, using existing data from sequencing projects and future sequencing data generated in clinical labs, might reveal interesting insights regarding the CNV aspect of the so-called missing heritability.

In the near future, the CNV field would benefit from (1) ongoing large sequencing projects like the 1000 Genomes to learn more about genome plasticity; (2) access to newer genotyping arrays that cover previously untagged SNPs; (3) developing open-access bioinformatics solution to facilitate and support clinical diagnosis; (4) establishing standards for clinical diagnosis and provide appropriate training to all the different players including physicians, biologists, and data analysts, and (5) further encouraging efforts on extensive phenotyping and data sharing between clinical and research labs.

## Conflict of Interest Statement

Armand Valsesia is a full-time employe at Nestlé Institute of Health Sciences SA. The funders had no role in preparation of this review or decision to publish.

## Footnotes

^1^http://www.ensembl.org/info/docs/variation/vep/index.html

^2^http://www.ncbi.nlm.nih.gov/clinvar/

^3^http://www.orpha.net/consor/cgi-bin/index.php?lng=EN

^4^http://www.hgvs.org/dblist/glsdb.html

^5^http://decipher.sanger.ac.uk/

^6^http://www.uk10k.org/

^7^https://www.ebi.ac.uk/ega/
